# A Lipid Nanoparticle-Formulated GP5-mRNA Vaccine Induces PRRSV-Specific Humoral and Cellular Immune Responses in BALB/c Mice

**DOI:** 10.3390/vetsci13090860

**Published:** 2026-08-25

**Authors:** Jia-Qi Zhang, Jian Huo, Lan-Lan Zheng, Shi-Jie Ma, Hui Hu, Zhong-Yi Fang, Yue Zhang, Hong-Ying Chen

**Affiliations:** 1 College of Veterinary Medicine, Henan Agricultural University, Zhengdong New District Longzi Lake 15#, Zhengzhou 450046, China; 2 Ministry of Education Key Laboratory for Animal Pathogens and Biosafety, Zhengdong New District Longzi Lake 15#, Zhengzhou 450046, China; 3 Henan Province Key Laboratory for Animal Food Pathogens Surveillance, Zhengzhou 450046, China; 4 Henan Institute of Agricultural and Animal Product Inspection and Technology, Zhengzhou 450008, China

**Keywords:** porcine reproductive and respiratory syndrome virus, GP5, mRNA vaccine, immunization

## Abstract

Porcine reproductive and respiratory syndrome virus (PRRSV) remains a major threat to the global swine industry, and the continuous emergence of genetically diverse strains poses challenges to vaccine development. In this study, a lipid nanoparticle-formulated mRNA vaccine encoding the PRRSV GP5 protein was constructed, and its preliminary immunogenicity was evaluated in BALB/c mice. The full-length ORF5 gene was amplified and cloned into the pIVT5 vector to generate the recombinant plasmid pIVT5-GP5. GP5-mRNA was synthesized via in vitro transcription and transfected into Marc-145 cells to verify antigen expression. Indirect immunofluorescence assay showed specific fluorescence signals in GP5-mRNA-transfected cells, and Western blotting further confirmed the expression of GP5 protein. The GP5-mRNA was subsequently encapsulated into lipid nanoparticles to prepare the GP5 mRNA-LNP vaccine formulation. BALB/c mice were immunized intramuscularly to evaluate humoral and cellular immune responses. The GP5 mRNA-LNP vaccine induced a GP5-specific IgG response, and sera collected on days 28, 35, and 42 after the initial immunization showed detectable neutralizing activity against PRRSV. Flow cytometric analysis showed an increased proportion of CD3^+^CD4^+^ and CD3^+^CD8^+^ T-cell subsets in the spleens of immunized mice. In addition, splenocytes from GP5 mRNA-LNP-immunized mice produced higher levels of IL-2, IFN-γ, and IL-4 after PRRSV antigen stimulation. Because mice are not the natural host of PRRSV, these results indicate that the GP5 mRNA-LNP vaccine candidate induced both humoral and cellular immune responses in BALB/c mice, providing a preliminary immunological basis for further optimization and evaluation in pigs.

## Simple Summary

Porcine reproductive and respiratory syndrome virus (PRRSV) is an important pathogen that causes severe economic losses in the swine industry. Current vaccines are useful but often provide limited protection against genetic diversity or newly emerging PRRSV strains. Therefore, new vaccine strategies are still needed. In this study, we developed an mRNA vaccine candidate encoding the PRRSV GP5 protein, which is an important viral envelope protein involved in immune responses. The GP5-mRNA was encapsulated into lipid nanoparticles to improve delivery and was tested in BALB/c mice. The vaccine candidate successfully expressed GP5 protein in cells and induced a PRRSV-specific antibody response in mice. Sera from immunized mice also showed detectable neutralizing activity. Furthermore, vaccinated mice exhibited increased CD4^+^ and CD8^+^ T-cell responses, as well as higher levels of immune-related cytokine production after antigen stimulation. These findings suggest that the GP5 mRNA-LNP vaccine candidate can induce both humoral and cellular immune responses in mice. Because mice are not the natural host of PRRSV, further immunization and PRRSV challenge studies are needed to evaluate protective efficacy, viremia reduction, clinical protection, and pathological changes in pigs.

## 1. Introduction

Porcine reproductive and respiratory syndrome (PRRS) remains one of the most economically devastating diseases affecting the swine industry worldwide since its first discovery in the United States in 1987 and in Europe in 1990 [1,2,3,4,5]. This disease is characterized by reproductive failure in sows and respiratory symptoms in pigs of different ages [6]. Porcine reproductive and respiratory syndrome virus (PRRSV), the etiological agent of PRRS, is an enveloped, positive-sense single-stranded RNA virus, which belongs to the family *Arteriviridae* within the order *Nidovirales* [7]. PRRSV is classified into PRRSV-1 and PRRSV-2, and PRRSV-2 remains predominant in China, where multiple lineages and variants, including highly pathogenic PRRSV (HP-PRRSV), NADC30-like, and NADC34-like strains, have circulated in recent years [8,9,10]. The high genetic variability, frequent mutation, and recombination of PRRSV make control efforts more challenging [8].

The PRRSV genome is approximately 15.4 kb in length and contains at least 10 open reading frames (ORFs), encoding 16 nonstructural proteins and 8 structural proteins (GP2, GP2b, GP3, GP4, GP5, 5a, M, and N) [11]. Among these structural proteins, GP5, encoded by ORF5, is the major envelope glycoprotein of PRRSV and is one of the most extensively studied antigens in PRRSV vaccine development. GP5 contains major antigenic epitopes, including neutralizing and non-neutralizing epitopes, and has been identified as an important target of virus-neutralizing antibodies [12,13,14]. Previous studies have also shown that GP5/ORF5 genetic variation is associated with differences in virus neutralization and cross-neutralization among PRRSV isolates [15]. Therefore, GP5 is a rational target antigen for evaluating PRRSV vaccine-induced antibody responses [16]. Although multi-structural-protein vaccines may provide broader antigenic coverage, GP5 was selected in the present study as a single-antigen proof-of-concept target due to its well-defined immunogenicity characteristics and its role as the major envelope glycoprotein involved in virus-neutralizing antibody responses.

Vaccination remains the primary strategy for prevention and control of PRRS. Currently available commercial PRRS vaccines mainly include inactivated vaccines and modified live vaccines. Inactivated vaccines are generally considered safer, but often induce limited cellular immune responses and provide insufficient protection, whereas modified live vaccines typically induce stronger immune responses but may raise concerns regarding viral shedding, recombination, residual virulence, or the risk of reversion to virulence [10,17]. More importantly, the protective effect of commercial PRRSV vaccines against homologous strains is generally superior to their effect against heterologous or newly emerging variants [10,17,18]. This limitation is particularly important for PRRSV, as the virus undergoes frequent mutation and recombination, leading to the emergence of multiple field strains, such as HP-PRRSV, NADC30-like, and NADC34-like variants [8,9,10]. Therefore, given the continuous evolution of PRRSV and the limitations of current vaccine-induced cross-protection, there is an ongoing need to explore alternative vaccine strategies that may better accommodate antigenic variation and improve immune response profiles.

In this context, mRNA vaccine platforms have attracted increasing attention in recent years. Compared with traditional vaccines, mRNA vaccines offer several potential advantages, including rapid design and production [19], flexible antigen optimization, and the ability to induce both humoral and cellular immune responses [20]. Because mRNA vaccines do not require the handling of infectious virus during antigen production and can be rapidly redesigned according to emerging viral sequences [19,21], they are particularly attractive for highly variable RNA viruses. Recent studies have highlighted broad application prospects of mRNA vaccines in preventing infectious diseases due to their safety and immunogenicity [22,23,24]. Among current delivery systems, lipid nanoparticles (LNPs) are the most widely used platform for mRNA vaccine delivery, owing to their ability to protect mRNA from degradation and facilitate its intracellular delivery [25,26]. These characteristics provide a basis for exploring LNP-formulated mRNA vaccines as a flexible strategy for PRRSV vaccine development.

In this study, ORF5 was selected as the target antigen gene, and an LNP-formulated GP5-mRNA vaccine candidate was prepared. The purpose of using a single GP5 antigen was to establish a preliminary proof-of-concept model to evaluate the immunogenicity of a PRRSV envelope-antigen mRNA-LNP vaccine, rather than claiming broad protection against genetically diverse PRRSV strains. The immunogenicity of the GP5 mRNA-LNP vaccine was systematically evaluated in a BALB/c mouse model using indirect Enzyme-Linked Immunosorbent Assay (ELISA), serum neutralization assays, flow cytometry and cytokine detection. This study provides preliminary experimental evidence for the immunogenicity of a PRRSV GP5 mRNA-LNP vaccine candidate and lays the foundation for further evaluation in pigs.

## 2. Materials and Methods

### 2.1. Cell, Plasmid and Virus

Marc-145 cells and plasmid pIVT5 were preserved in our laboratory. Marc-145 cells were cultured in Dulbecco’s Modified Eagle Medium (DMEM, Thermo Fisher Scientific, Shanghai, China) supplemented with 10% fetal bovine serum (FBS) (Gibco, Carlsbad, CA, USA), 100 U/mL penicillin and 100 μg/mL streptomycin. The PRRSV HeN-HC strain, an HP-PRRSV type strain isolated and stored in our laboratory [27], was propagated in Marc-145 cells and used for antigen preparation and serum neutralization assays.

All experimental procedures were reviewed and approved by the Henan Agriculture University Animal Care and Use Committee (approval code: HNND2025010511; approval date: 5 January 2025).

### 2.2. Construction of Plasmid pIVT5-GP5 and In Vitro Transcription of GP5-mRNA

**Figure 1:**
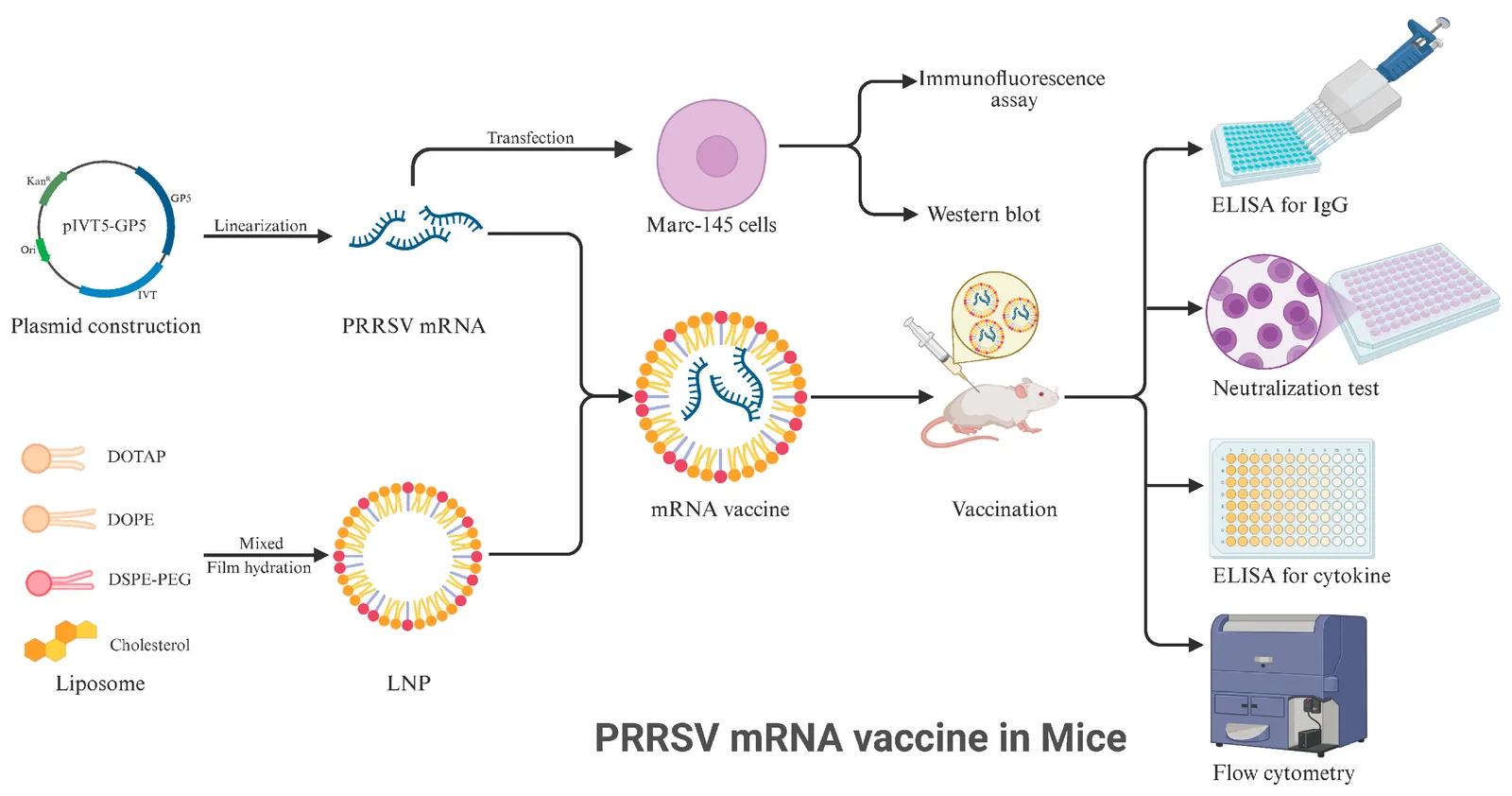
The overall process. Firstly, a plasmid pIVT5-GP5 harboring the GP5 gene was constructed, purified, and linearized. Then, GP5-mRNA was transcribed in vitro, and cellular expression was validated by indirect immunofluorescence assay and Western blotting analysis. LNPs were prepared, and GP5-mRNA was encapsulated with LNPs to form a complete mRNA vaccine. The mRNA vaccine was given to mice and evaluated by ELISA for IgG, a neutralization test, ELISA for cytokines, and flow cytometry.

The full-length GP5 gene of PRRSV was amplified from viral cDNA using the specific primers GP5-F (GGATTCATGTTGGGGAAGTGCTTGACC) and GP5-R (AAGCTTCTAGAGACGACCCCATTGTTCC). PCR was performed using 2×Rapid Taq Master Mix (Vazyme Biotechnology Co., Ltd., Nanjing, China) under the following conditions: 95 °C for 3 min; 39 cycles of 95 °C for 15 s, 58 °C for 15 s, and 72 °C for 20 s; followed by 72 °C for 10 min. The purified PCR product was digested by restriction enzymes *Bam* HI and *Hin*d III and inserted into the similarly digested plasmid pIVT5. The resulting recombinant plasmid was transformed into *Escherichia coli* DH5α competent cells (Takara, Dalian, China) and verified by PCR, restriction enzyme digestion, and sequencing, then named pIVT5-GP5 (Figure 1).

For in vitro transcription, plasmid pIVT5-GP5 was linearized with restriction enzyme *Sal* I, dephosphorylated using alkaline phosphatase (CIAP), and purified. GP5-mRNA was synthesized using a T7 High Yield RNA Synthesis Kit (Synthgene Biotechnology Co., Ltd., Nanjing, China, C30112) following the manufacturer’s protocol. After transcription at 37 °C for 3 h, residual DNA template was removed by RNase-free DNase treatment at 37 °C for 15 min. The synthesized mRNA products were purified by lithium chloride precipitation, dissolved in RNase-free water, quantified using a NanoDrop spectrophotometer (Thermo Fisher Scientific, Waltham, MA, USA), and stored at −80 °C until further use.

### 2.3. In Vitro Expression of GP5-mRNA

The Marc-145 cells were seeded into 6-well plates and transfected with GP5-mRNA when the cells reached 70% confluency. For each well, 4 μg of GP5-mRNA was mixed with Opti-MEM and Lipofectamine^TM^ 3000 reagent (Thermo Fisher Scientific) according to the manufacturer’s protocol. After 5 h of transfection, the medium was replaced with fresh culture medium. Mock-transfected cells were used as controls.

At 24 h post-transfection, GP5 expression was detected by indirect immunofluorescence assay (IFA). Briefly, cells were fixed with paraformaldehyde for 15 min, permeabilized with 0.5% Triton X-100 (9002-93-1, Sangon Biotech Co., Ltd., Shanghai, China) for 20 min, and incubated with a rabbit anti-GP5 polyclonal antibody (bs-4504R, Bioss, Beijing, China) for 1 h at 37 °C, followed by Fluorescein (FITC)-conjugated goat anti-rabbit IgG (SA00003-2, Proteintech Group, Inc., Wuhan, China) and DAPI (0.5 µg/mL, Beyotime, Shanghai, China) staining for 15 min, then washed with PBS three times. Fluorescence was observed under an inverted fluorescence microscope.

At 48 h post-transfection, the cells were collected for Western blotting. Cell lysates were separated by 15% SDS-PAGE and transferred onto PVDF membranes (Millipore, Burlington, MA, USA). The membrane was blocked with 5% non-fat milk and incubated overnight at 4 °C with rabbit anti-GP5 protein serum (1:200, produced by our laboratory, unpublished data), followed by horseradish peroxidase (HRP)-goat anti-rabbit IgG (1:5000, Proteintech, Wuhan, China). Protein bands were visualized using Omni-ECL^TM^ Enhanced Chemiluminescence Kit HRP (Epizyme, Shanghai, China).

### 2.4. Preparation of GP5 mRNA-LNP Vaccine

The GP5 mRNA-LNP vaccine was prepared by thin-film hydration and extrusion as described previously [28]. Briefly, DOTAP, cholesterol, DOPE, and DSPE-PEG were dissolved in chloroform at a mass ratio of 3.7:2.49:1:1. The chloroform was removed using a rotary evaporator until a uniform lipid film formed on the bottle wall, followed by flushing with nitrogen to avoid lipid oxidation. GP5-mRNA was added at a mass ratio of 2:1 and mixed at 42 °C. The formulation was sequentially extruded through 400-nm and 200-nm membranes to obtain the GP5 mRNA-LNP vaccine. Particle size and zeta potential were measured using a nanoparticle size and zeta potential analyzer.

### 2.5. Mouse Immunization

A total of 60 six-week-old specific-pathogen-free (SPF) BALB/c mice with an average body weight of 18 g were randomly allocated into three groups, with 20 mice per group. Mice were intramuscularly immunized with the GP5 mRNA-LNP vaccine, a commercial PRRSV attenuated live vaccine (KeLanNing, Wuhan, China), or DMEM as the negative control. Immunizations were administered on days 0, 14, and 28. The commercial vaccine was prepared according to the manufacturer’s instructions, with each mouse receiving 100 μL. The GP5 mRNA-LNP vaccine group, commercial vaccine group, and DMEM control group followed the same immunization protocol. Blood samples were collected via the tail vein of mice on days 14, 28, 35 and 42 after the initial immunization. Serum samples were separated by centrifugation and stored at −80 °C until further use. Sera collected at these time points were used for serum neutralization assays, whereas sera collected on day 42 were also used for indirect ELISA detection of endpoint GP5-specific IgG. On day 42, all mice were euthanized, and spleens of five mice from each group were taken for flow cytometric analysis and cytokine detection.

### 2.6. Indirect ELISA

GP5-specific IgG antibodies in the serum of mice on day 42 were detected by indirect ELISA. Briefly, 96-well plates were coated overnight at 4 °C using purified GP5 protein (400 ng per well) and blocked with 1% bovine serum albumin (BSA, Invitrogen, Carlsbad, CA, USA) at 37 °C for 2 h. Serially diluted serum samples were added and incubated at 37 °C for 1 h. After washing, HRP-conjugated goat anti-mouse IgG (H+L) (1:6000, Proteintech, Wuhan, China) was added and incubated at 37 °C for 1 h. TMB substrate solution was used for color development, and the reaction was stopped with 2 M H_2_SO_4_. The optical density (OD) was measured at 450 nm using a microplate reader.

### 2.7. Serum Neutralization Assay

Serum neutralizing antibodies against PRRSV were detected using a microtiter serum neutralization assay. Sera were collected on days 14, 28, 35, and 42 after the initial immunization and used for this assay. Briefly, Marc-145 cells were seeded into 96-well plates and cultured until the cell density reached 85–90%. Heat-inactivated sera were initially diluted at 1:2 and then serially diluted two-fold. Equal volumes of diluted sera and PRRSV HeN-HC suspension were mixed to achieve a final virus input of 100 TCID_50_/well. After incubation at 37 °C for 1 h, the serum–virus mixtures were added to Marc-145 cell monolayers, with eight replicate wells for each serum dilution. Each plate included virus control wells containing PRRSV without serum and cell control wells without PRRSV. Cells were incubated until an obvious PRRSV-induced cytopathic effect (CPE) appeared in the virus control wells, while the cell control wells remained intact. CPE of each well was scored by comparison with the virus control and cell control. The neutralizing antibody titer was defined as the reciprocal of the highest serum dilution at which at least 50% of replicate wells showed inhibition of PRRSV-induced CPE. The 50% endpoint titer was determined using the Reed–Muench method based on the CPE-positive and CPE-negative results across serial dilutions.

### 2.8. Immune Cell Phenotyping

On day 42 after the initial immunization, spleens were collected from five mice in each group. Single-cell suspensions were prepared by passing spleen tissues through a 70-μm cell filter and then performing red blood cell lysis. The splenocyte suspensions were washed and resuspended in PBS (4 × 10^5^ cells). Cells were stained with FITC Rat Anti-Mouse CD8a (catalog No. 553030), FITC Rat Anti-Mouse CD4 (catalog No. 553055), and APC Hamster Anti-Mouse CD3e (catalog No. 561826) antibodies (BD Biosciences Pharmingen, CA, USA) at 4 °C for 30 min in the dark. Because CD4 and CD8 antibodies were both FITC-conjugated, CD4 and CD8 staining was performed in separate tubes, with each tube co-stained with APC-conjugated anti-CD3e antibodies. After washing, samples were analyzed by flow cytometry. Debris and dead-cell-enriched events were excluded based on forward scatter and lateral scatter properties, and lymphocyte-enriched events were used as the parent population for analysis. Positive gates for CD3, CD4, and CD8 were established using the available unstained/negative control and then applied consistently to the analyzed samples. Viability dye staining and Fc receptor blocking with anti-CD16/32 antibody were not performed before staining.

### 2.9. Cytokine Detection

Splenocytes collected from the GP5 mRNA-LNP vaccine, commercial vaccine, and DMEM-immunized groups on day 42 were resuspended at 5 × 10^6^ cells/well and seeded in 24-well plates. Splenocytes were stimulated with purified PRRSV antigen at a final concentration of 2 μg/mL for 24 h, while complete RPMI medium without PRRSV antigen was used as the in vitro unstimulated control. After incubation, culture supernatants were harvested. The levels of mouse IFN-γ (catalog No. 558258), IL-2 (catalog No. 555148), and IL-4 (catalog No. 555232) were measured using commercial sandwich ELISA kits (Biosource, Camarillo, CA, USA) following the manufacturer’s protocol.

### 2.10. Statistical Analysis

Data were analyzed using GraphPad Prism 9.5 and are presented as mean ± standard deviation (SD) or mean ± standard error of the mean (SEM), as indicated in the figure legends. Comparisons among multiple groups were performed using one-way ANOVA followed by Tukey’s multiple-comparisons test (* *p* < 0.05, ** *p* < 0.01, and *** *p* < 0.001).

## 3. Results

### 3.1. Construction of the PRRSV GP5 mRNA-LNP Vaccine Platform

To develop a PRRSV mRNA vaccine candidate, the full-length GP5 gene of PRRSV HeN-HC was amplified and cloned into the pIVT5 vector to generate the recombinant plasmid pIVT5-GP5. After plasmid verification and linearization, GP5-mRNA was synthesized by in vitro transcription and subsequently encapsulated into lipid nanoparticles. The overall workflow included GP5-mRNA production, in vitro antigen-expression validation, LNP formulation, mouse immunization, and evaluation of humoral and cellular immune responses (Figure 1).

### 3.2. In Vitro Expression of GP5-mRNA in Marc-145 Cells

**Figure 2:**
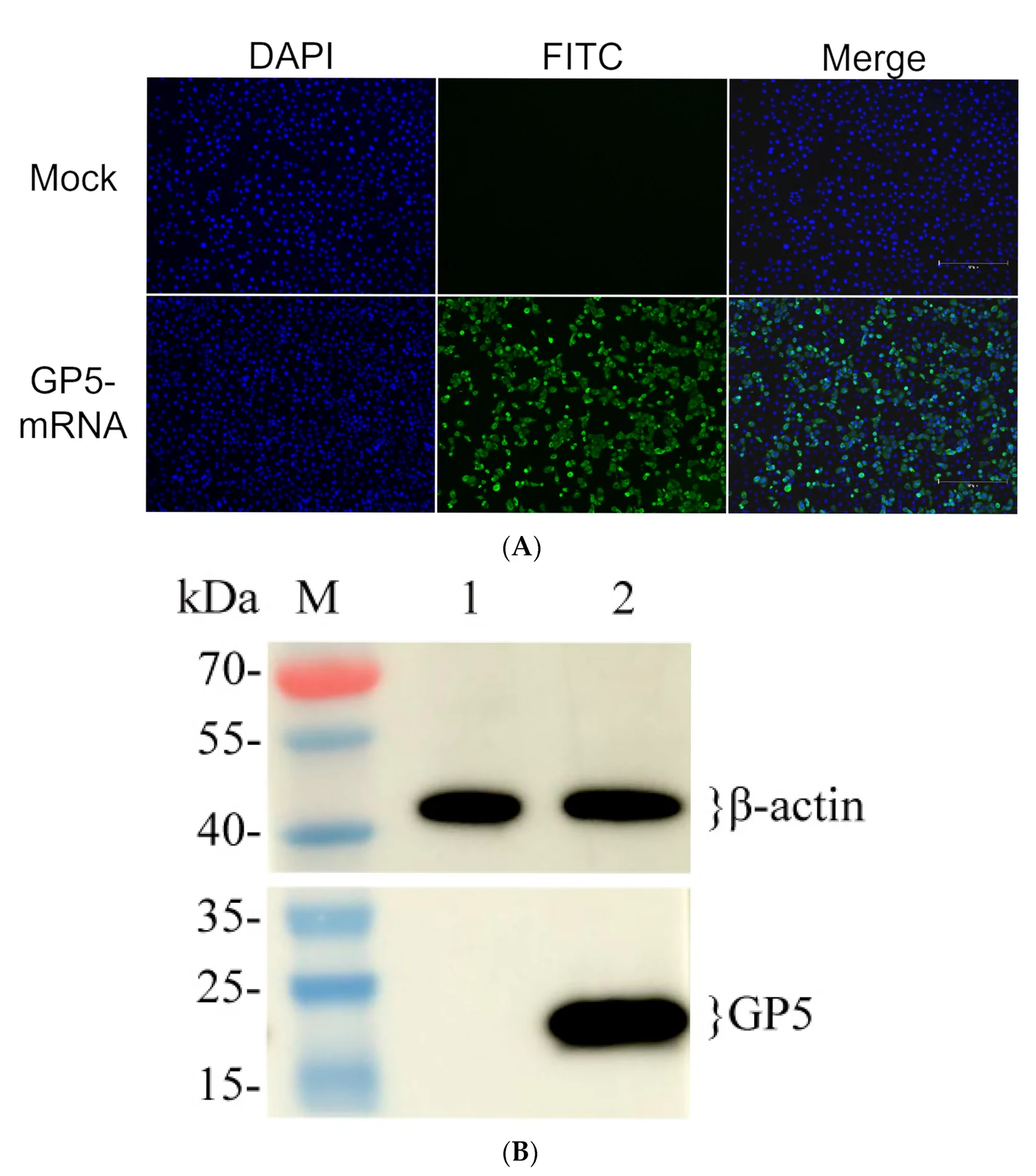

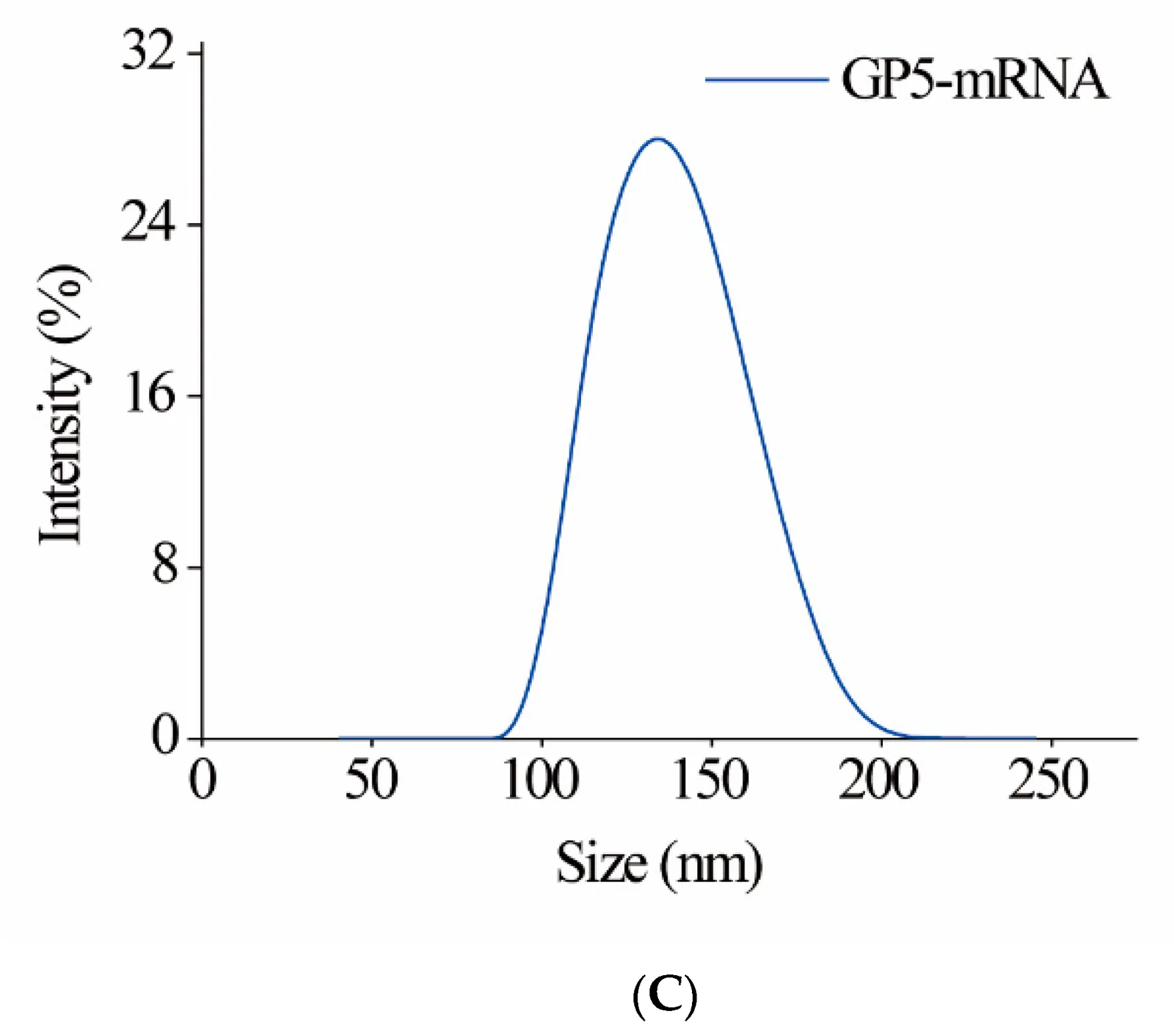
Identification of PRRSV GP5 mRNA-LNP vaccine. (**A**) Expression of mRNA in Marc-145 cells was analyzed by IFA. GP5 protein was labeled with green fluorescence, and cell nuclei were counterstained with DAPI (blue). (**B**) Expression of mRNA in Marc-145 cells was analyzed by Western blotting. M, protein molecular weight marker; lane 1, mock-transfected cells; lane 2, GP5-mRNA-transfected cells. The uncropped Western blot images are provided in Supplementary Figure S1. (**C**) Particle size of the mRNA-LNPs composed of DOTAP, cholesterol, DOPE, and DSPE-PEG was measured using dynamic light scattering (DLS).

To determine whether the synthesized GP5-mRNA could express the target antigen, Marc-145 cells were transfected with GP5-mRNA and examined by IFA and Western blotting. Specific green fluorescence was observed in GP5-mRNA-transfected cells, whereas no specific fluorescence was detected in mock-transfected cells (Figure 2A). Western blotting analysis further demonstrated that a GP5-specific band of approximately 25 kDa was detected in GP5-mRNA-transfected cells, but not in the mock; meanwhile, β-actin as an internal control was detected in both groups (Figure 2B). These results confirmed the successful expression of GP5 protein by GP5-mRNA in Marc-145 cells.

### 3.3. Characterization of GP5 mRNA-LNP Vaccine

GP5-mRNA was encapsulated with LNPs to generate the GP5 mRNA-LNP vaccine. Nanoparticle analysis showed that the formulation displayed a main particle size distribution peak at approximately 135 nm, with a zeta potential of approximately 25 mV (Figure 2C). These data indicated that GP5-mRNA was successfully formulated into an LNP-based vaccine for subsequent mouse immunization.

### 3.4. GP5 mRNA-LNP Vaccine Induced GP5-Specific IgG Responses in Mice

**Figure 3:**
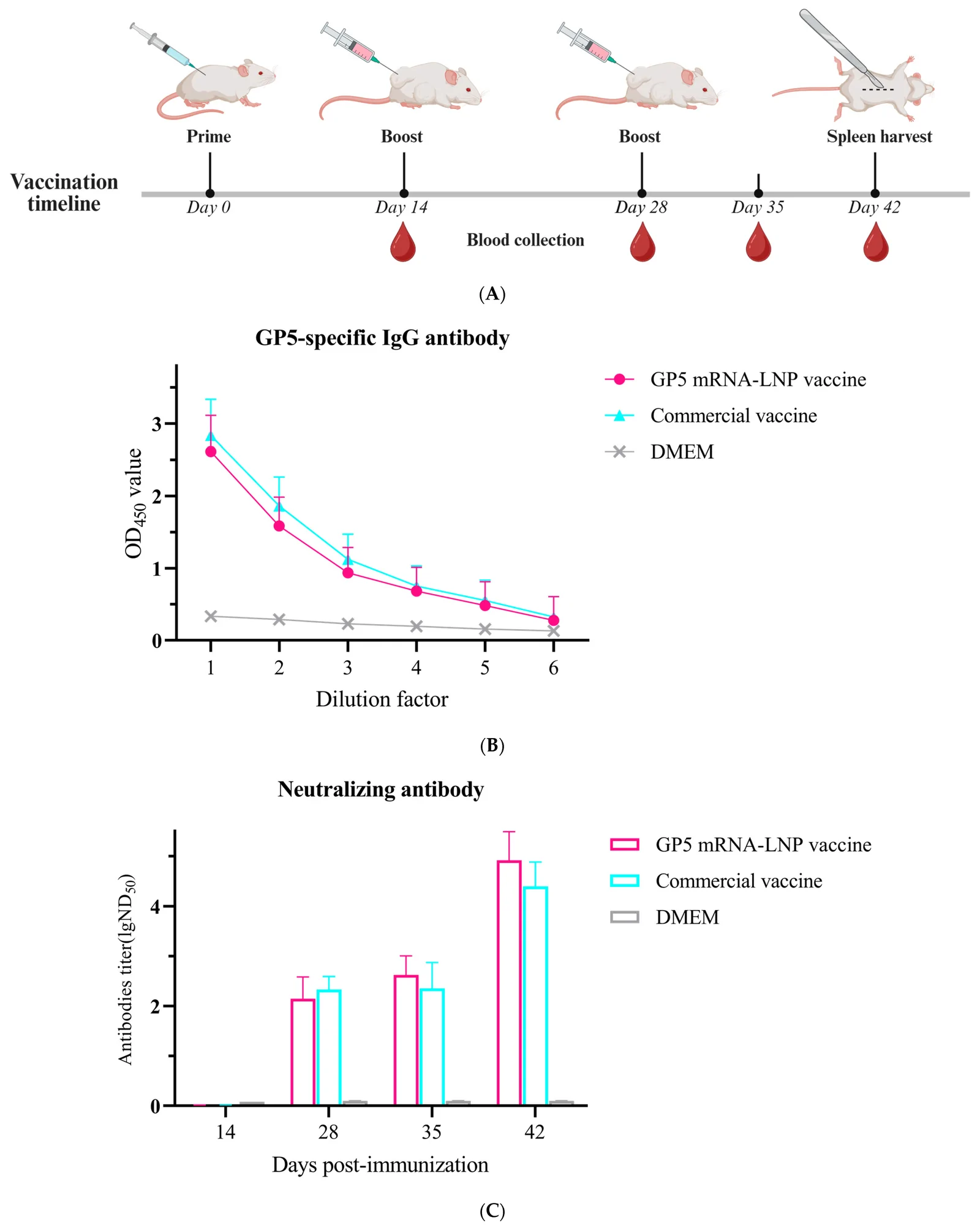
Humoral immune responses induced by the GP5 mRNA-LNP vaccine in mice. (**A**) Immunization schedule and sample collection timeline. BALB/c mice (n = 20/group) were intramuscularly injected with GP5 mRNA-LNP vaccine, commercial PRRSV attenuated live vaccine, or DMEM on days 0, 14 and 28. Blood samples were collected on day 14, day 28, day 35 and day 42, and mice were euthanized on day 42 for spleen collection. (**B**) GP5-specific IgG antibody responses were measured by indirect ELISA using sera collected on day 42. Each serum was serially diluted from 1:200 to 1:25,600, with dilution factors of 1–6, respectively. (**C**) Sera collected on days 14, 28, 35, and 42 were analyzed for neutralizing antibody titers against the homologous PRRSV HeN-HC strain. Data are one representative result of two independent experiments and are presented as the mean ± SEM.

BALB/c mice were immunized intramuscularly with the GP5 mRNA-LNP vaccine, commercial PRRSV attenuated live vaccine, or DMEM on days 0, 14, and 28, and blood samples were collected according to the immunization schedule (Figure 3A). To evaluate the endpoint GP5-specific antibody response, sera collected on day 42 were analyzed by indirect ELISA. Mice immunized with the GP5 mRNA-LNP vaccine developed higher levels of GP5-specific IgG compared to the DMEM control group (Figure 3B). At serial serum dilutions, the OD_450_ values of the GP5 mRNA-LNP vaccine group were higher than those of the control group, indicating that GP5 mRNA-LNP vaccination induced a detectable antigen-specific humoral immune response in mice.

### 3.5. GP5 mRNA-LNP Vaccine Induced Serum Neutralizing Activity

The functional antibody response was further evaluated by a serum neutralization assay using PRRSV HeN-HC and Marc-145 cells. Sera collected on days 14, 28, 35, and 42 after the initial immunization were used for neutralization analysis. No detectable neutralizing activity was observed in the DMEM control group. In contrast, sera from GP5 mRNA-LNP-vaccinated mice showed detectable neutralizing activity from day 28 onward, with neutralizing antibody titers increasing over time (Figure 3C). Neutralizing activity was more evident at later time points, especially on day 42 after the initial immunization. These results indicated that the GP5 mRNA-LNP vaccine induced not only GP5-specific binding antibodies in mice, but also functional antibodies with neutralizing activity against the homologous PRRSV HeN-HC strain. Representative images of PRRSV-induced CPE and CPE inhibition are provided in Supplementary Figure S2. However, neutralizing activity against heterologous PRRSV strains was not evaluated in this study.

### 3.6. GP5 mRNA-LNP Vaccine Promoted T-Cell Responses in Mice

**Figure 4:**
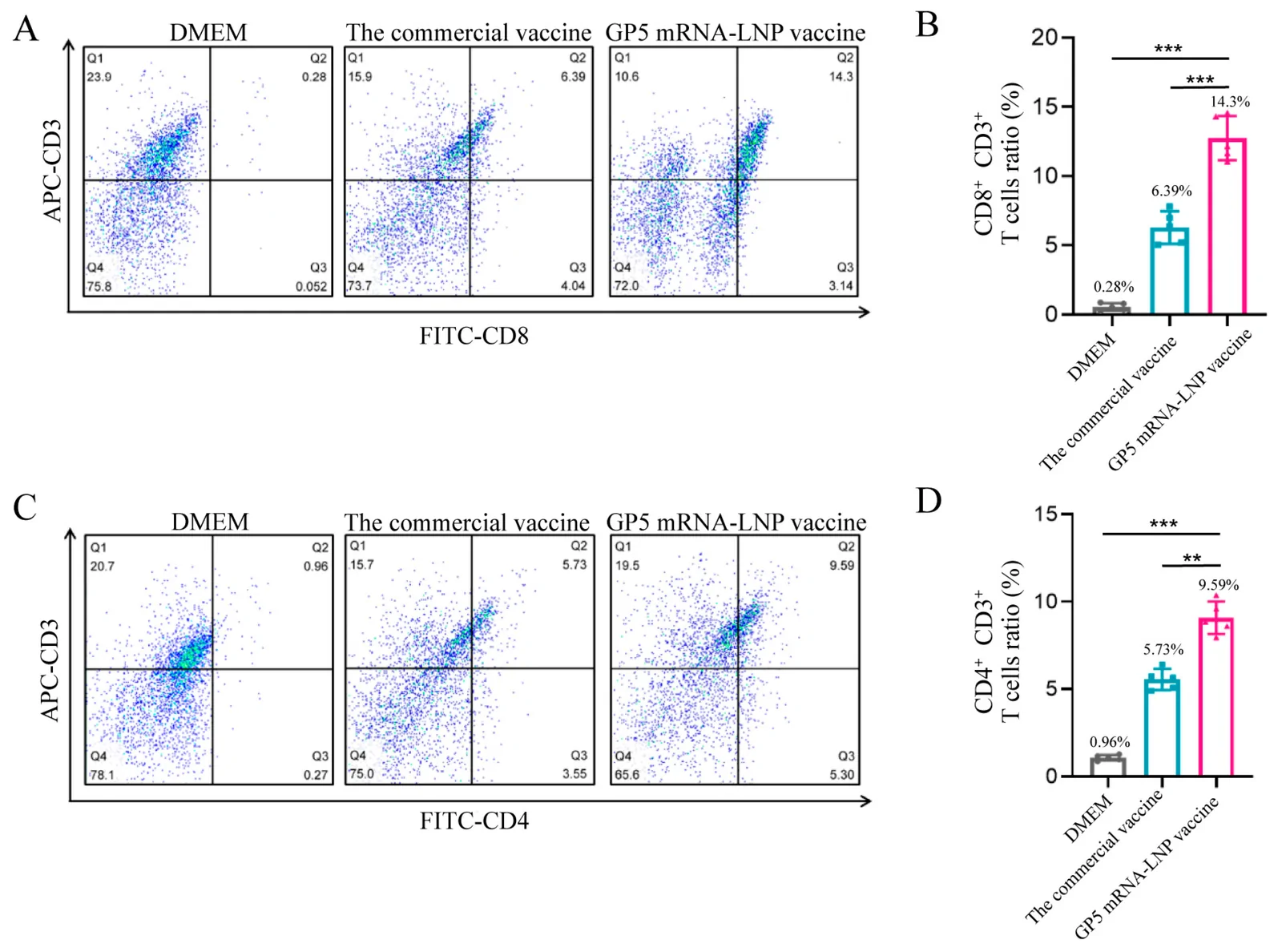
Cellular immune responses induced by the GP5 mRNA-LNP vaccine in mice. On day 42 after the initial immunization, mouse spleens were harvested, and splenocytes were isolated for flow cytometric analysis. Lymphocyte-enriched events were selected by FSC/SSC gating. The percentages of CD3^+^CD4^+^ and CD3^+^CD8^+^ T cells were calculated among lymphocyte-enriched events. (**A**) Representative flow cytometry plots of CD3^+^CD8^+^ T cells. (**B**) Quantification of CD3^+^CD8^+^ T-cell proportions. (**C**) Representative flow cytometry plots of CD3^+^CD4^+^ T cells. (**D**) Quantification of CD3^+^CD4^+^ T-cell proportions. The available FSC/SSC lymphocyte gate and unstained/negative control used for gate setting are provided in Supplementary Figure S3. Data are presented as mean ± SEM. Statistical significance was analyzed using one-way ANOVA followed by Tukey’s multiple-comparisons test. ** *p* < 0.01 and *** *p* < 0.001.

To evaluate cellular immune responses, splenocytes were collected from immunized mice on day 42 and analyzed by flow cytometry. Compared with the DMEM control group, the GP5 mRNA-LNP vaccine group showed an increase in the proportion of both CD3^+^CD4^+^ and CD3^+^CD8^+^ T cells (Figure 4). The proportions of CD3^+^CD8^+^ T cells in the DMEM control, commercial vaccine, and GP5 mRNA-LNP vaccine groups were 0.28%, 6.39%, and 14.3%, respectively. The proportions of CD3^+^CD4^+^ T cells were 0.96%, 5.73%, and 9.59%, respectively. These results suggested that GP5 mRNA-LNP vaccination promoted T-cell immune responses in mice.

### 3.7. GP5 mRNA-LNP Vaccine Induced Cytokine Responses in Splenocytes

**Figure 5:**
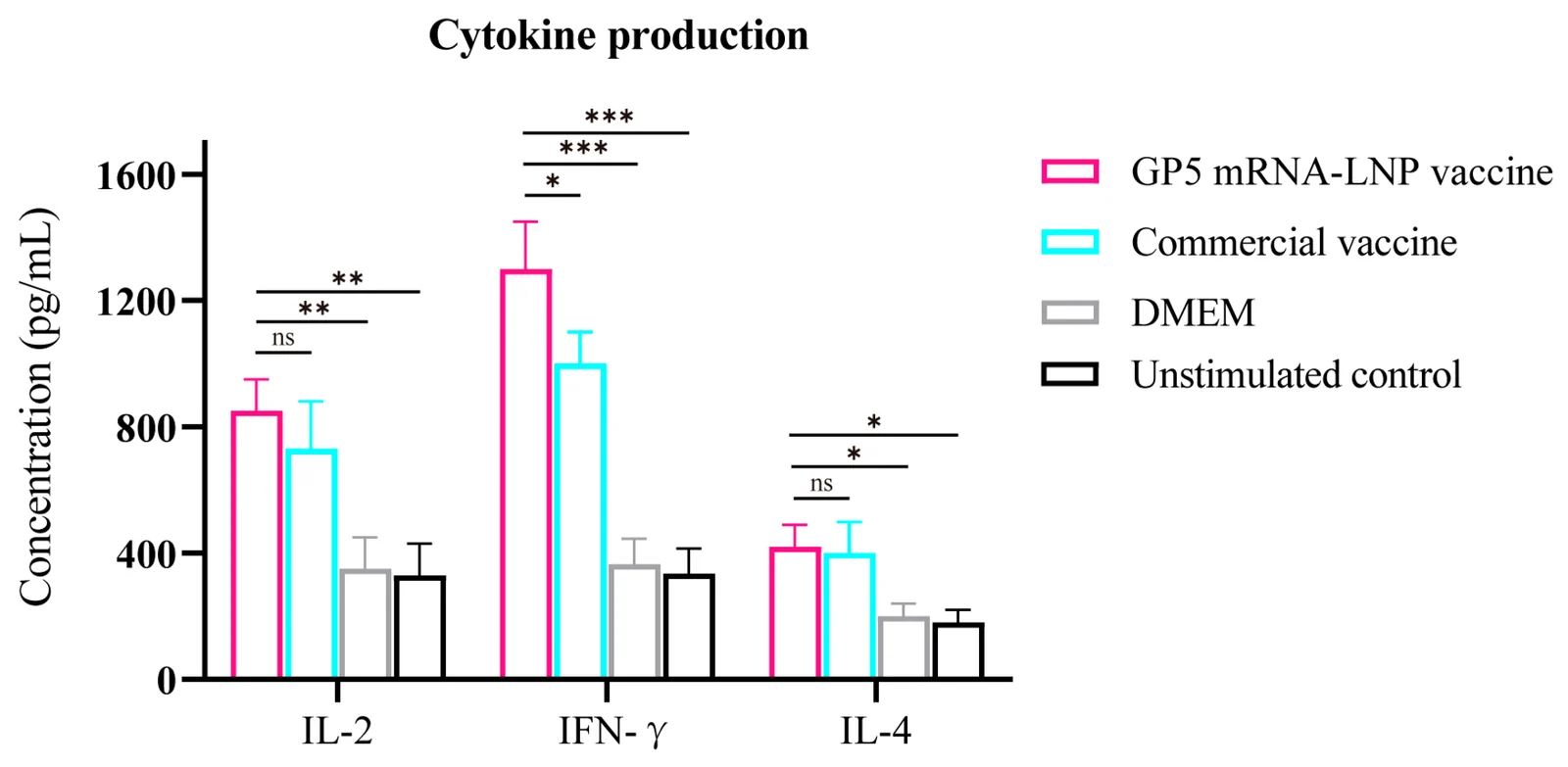
Cytokine production by splenocytes from immunized mice after PRRSV antigen stimulation. Splenocytes were collected from immunized mice on day 42 after the initial immunization. Splenocytes from the GP5 mRNA-LNP vaccine, commercial vaccine, and DMEM-immunized groups were stimulated with purified PRRSV antigen at a final concentration of 2 μg/mL for 24 h, while complete RPMI medium without PRRSV antigen was used as the in vitro unstimulated control. DMEM indicates the DMEM-immunized mouse control group, whereas unstimulated control indicates splenocytes cultured with complete RPMI medium only. IL-2, IFN-γ, and IL-4 levels were analyzed separately using one-way ANOVA, followed by Tukey’s multiple-comparisons test. Data are presented as mean ± SD. ns, not significant; * *p* < 0.05, ** *p* < 0.01 and *** *p* < 0.001.

To evaluate antigen-specific cytokine responses, splenocytes collected on day 42 were stimulated with purified PRRSV antigen, and cytokine production was measured by ELISA. Complete RPMI medium without PRRSV antigen was used as the in vitro unstimulated control. Compared with the DMEM-immunized control group and the unstimulated control, splenocytes from the GP5 mRNA-LNP vaccine-immunized mice produced higher levels of IFN-γ, IL-2, and IL-4 after antigen stimulation (Figure 5). The IL-2 and IL-4 levels in the GP5 mRNA-LNP group were comparable to those in the commercial vaccine group, whereas the IFN-γ level in the GP5 mRNA-LNP vaccine group was significantly higher than that in the commercial vaccine group.

## 4. Discussion

PRRSV remains a major challenge for swine health because of its high genetic variability, frequent mutation, and recombination, which reduce the cross-protective efficacy of current vaccines against heterologous or newly emerging strains [1,29,30,31,32]. Although commercial modified live and inactivated vaccines are widely used, their performance against homologous strains is often stronger than against antigenically diverse field strains. In this context, mRNA vaccines provide a flexible platform for rapid antigen design and sequence updating, while LNP delivery can protect mRNA from degradation and facilitate intracellular delivery [17,18,19,21,25]. In the present study, we constructed an LNP-formulated GP5-mRNA vaccine candidate and evaluated its preliminary immunogenicity in BALB/c mice.

GP5 was selected as the target antigen because it is the main envelope glycoprotein of PRRSV and contains important antigenic epitopes associated with virus-specific immune responses. Previous studies have identified neutralizing and non-neutralizing epitopes in the GP5 ectodomain, suggesting that GP5 can serve as a target for homologous and, in some cases, broadly neutralizing antibodies [13,14]. In this study, GP5-mRNA expression was confirmed in Marc-145 cells by immunofluorescence assay and Western blotting, and the GP5-mRNA was formulated into LNPs with a main particle size of approximately 135 nm and a positive zeta potential. These results indicate that the GP5 mRNA-LNP vaccine candidate was successfully prepared for subsequent immunogenicity evaluation. However, GP5 is also one of the most variable structural proteins of PRRSV, and variation in the ORF5 sequence is related to the differences in virus neutralization and cross-neutralization among PRRSV isolates [15]. Therefore, a single-GP5 vaccine may have limited immune breadth against genetically diverse PRRSV strains.

Humoral immunity is an important component of vaccine-induced immune responses against PRRSV. GP5 mRNA-LNP vaccination induced detectable GP5-specific IgG responses in mice, indicating that the LNP-formulated mRNA vaccine was capable of expressing GP5 antigen and triggering antigen-specific antibody production. Serum neutralization assays further showed that sera collected at later time points, particularly on day 42 after the initial immunization, exhibited detectable neutralizing activity against the homologous PRRSV HeN-HC strain [13,14]. However, the neutralizing antibody response was still relatively low, and neutralization was evaluated only against the homologous strain. Therefore, the cross-neutralizing activity of the GP5 mRNA-LNP vaccine against heterologous PRRSV strains (including prevalent NADC30-like, NADC34-like, or other PRRSV-2 lineages) remains to be determined. Further optimization may be necessary, such as antigen sequence modification, dose adjustment, repeated boosting, or combination with other PRRSV structural proteins, to enhance the amplitude and breadth of neutralizing antibodies.

Cell-mediated immunity is also considered important for PRRSV control, particularly since individual antibody responses are not always correlated with protection. Flow cytometric analysis showed that GP5 mRNA-LNP-immunized mice had increased proportions of splenic CD3^+^CD4^+^ and CD3^+^CD8^+^ T-cell subsets compared with the control group. After stimulation with purified PRRSV antigen, splenocytes from GP5 mRNA-LNP-immunized mice produced elevated levels of IL-2, IFN-γ, and IL-4. IL-2 is associated with T-cell expansion and immune regulation [33], IFN-γ is typically related to antiviral cellular immunity [34], and IL-4 is generally associated with humoral immune-associated responses. The increased production of these cytokines suggests that GP5 mRNA-LNP vaccination induced both cellular and humoral immune-associated cytokine responses in BALB/c mice. Nevertheless, as the present study evaluated cytokine secretion in culture supernatants rather than single-cell functional responses, the antigen-specific T-cell phenotype induced by GP5 mRNA-LNP vaccination requires further characterization. Future studies using intracellular cytokine staining, ELISpot, lymphocyte proliferation assays, or activation-marker analysis will help elucidate the functional characteristics of GP5-specific T-cell responses [35].

Several limitations should be noted. Firstly, the immunogenicity of the GP5 mRNA-LNP vaccine was evaluated only in mice. PRRSV cannot replicate efficiently in rodents [36] because mice are not the natural hosts of PRRSV. Therefore, the present study assessed preliminary immunogenicity, rather than protective efficacy against PRRSV infection. The immune responses observed in BALB/c mice may not fully predict vaccine performance in pigs because of species differences in antigen presentation, lymphocyte responses, cytokine regulation, and virus–host interactions. Secondly, this study did not include pig immunization, viral challenge, viremia monitoring, viral shedding detection, tissue viral load analysis, or pathological evaluation. Moreover, the current mRNA-LNP vaccine candidate only contains one GP5 antigen. Given the high genetic variability of GP5 among PRRSV strains, immune escape and limited cross-protection against heterologous field strains may occur. Therefore, in the future, there may be a need for multivalent or multi-structural-protein mRNA vaccine designs to expand immune coverage against circulating PRRSV variants. Thirdly, the in vivo biosafety, degradation, and tissue distribution of the DOTAP-based LNP formulation have not been evaluated. As a cationic lipid, DOTAP may be associated with dose-dependent cytotoxicity, local reactivity, or inflammatory responses, and systematic safety assessment is required before further application [37]. An empty LNP-only control group was not included in this study; therefore, the potential contribution of the LNP vehicle to baseline innate or inflammatory responses cannot be completely ruled out and should be evaluated in future studies. From the perspective of field application in the pig industry, future studies should also compare optimized mRNA-LNP vaccine formulations with commercially available PRRSV vaccines in pigs and evaluate simplified immunization schedules, production cost, safety, and protection against multiple circulating PRRSV lineages. In addition, complete exported representative gating plots for all treatment groups were not available. Although lymphocyte-enriched events were selected by FSC/SSC gating and positive gates were established using the available unstained/negative control, viability dye staining and Fc receptor blocking were not performed. Therefore, the possibility that dead cells or nonspecific antibody binding affected the accuracy of T-cell subset quantification cannot be completely excluded. Future studies should include viability staining, Fc receptor blocking, and complete gating controls to improve the rigor of immune cell phenotyping.

## 5. Conclusions

In this study, an LNP-formulated GP5-mRNA vaccine candidate was successfully constructed and evaluated in BALB/c mice. The vaccine expressed GP5 protein in vitro and induced GP5-specific IgG responses, detectable homologous serum neutralizing activity, increased splenic CD3^+^CD4^+^ and CD3^+^CD8^+^ T-cell subsets, and enhanced IL-2, IFN-γ, and IL-4 production after PRRSV antigen stimulation. These findings indicate that the GP5 mRNA-LNP vaccine candidate can induce both humoral and cellular immune responses in mice. Because mice are not the natural host of PRRSV, further optimization and evaluation in pigs are required to determine its protective efficacy and practical application potential.

## Data Availability

The original contributions presented in this study are included in the article. Further inquiries can be directed to the corresponding author.

## Supplementary Materials

The following supporting information can be downloaded at: https://www.mdpi.com/article/10.3390/vetsci13090860/s1, Figure S1: Uncropped Western blot images; Figure S2: Representative images of PRRSV-induced CPE and CPE inhibition in Marc-145 cells; Figure S3: Available flow cytometry gating information for splenic T-cell subset analysis.
