# Supplementary material for: A Lipid Nanoparticle-Formulated GP5-mRNA Vaccine Induces PRRSV-Specific Humoral and Cellular Immune Responses in BALB/c Mice
Source: Vet Sci. 2026 Aug 25;13(9):860. doi: 10.3390/vetsci13090860 (PMC13611864; doi:10.3390/vetsci13090860)
Supplement: Supplementary file 1 [file vetsci-13-00860-s001.zip › vetsci-4464153-supplementary.pdf]

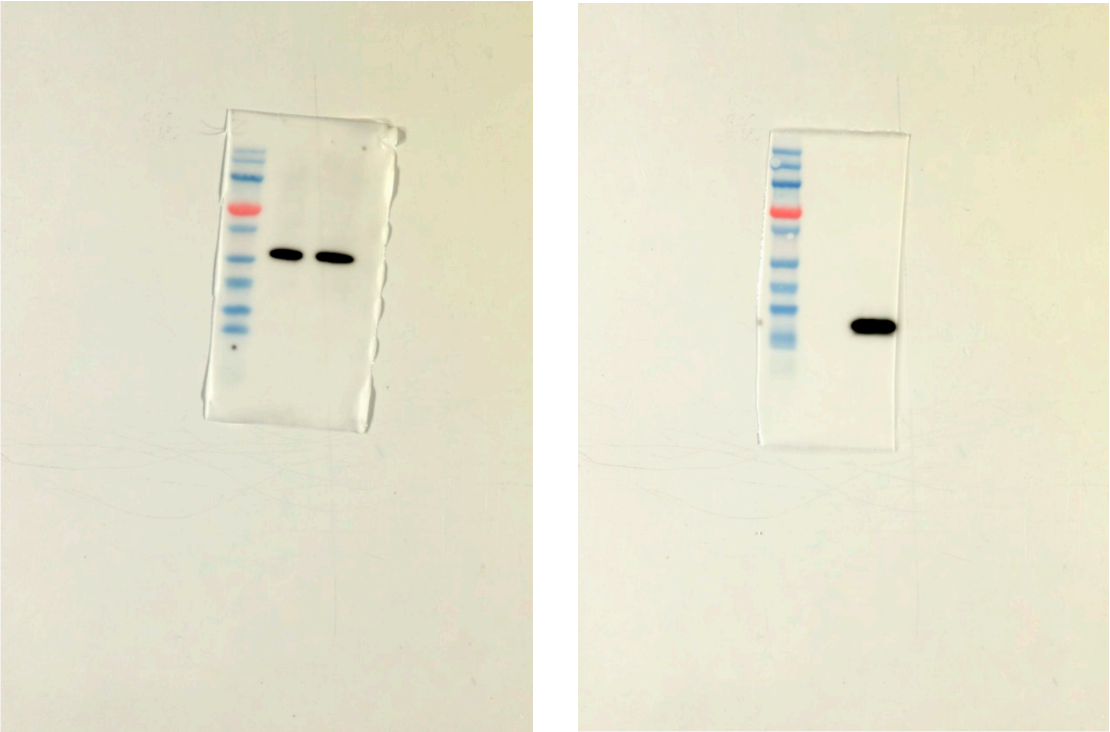

Supplementary Figure S1. Uncropped Western blot images.

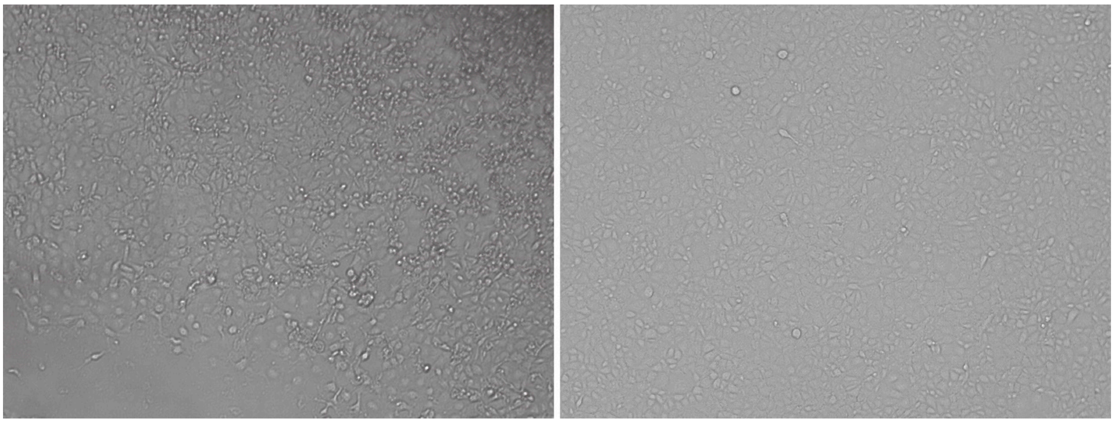

Supplementary Figure S2. Representative images of PRRSV-induced CPE and CPE inhibition in Marc-145 cells. Marc-145 cells infected with PRRSV HeN-HC in the absence of serum showed typical cytopathic effect, whereas cells incubated with neutralizing serum-virus mixtures showed inhibition of PRRSV-induced CPE. Cell control wells without PRRSV infection remained intact.

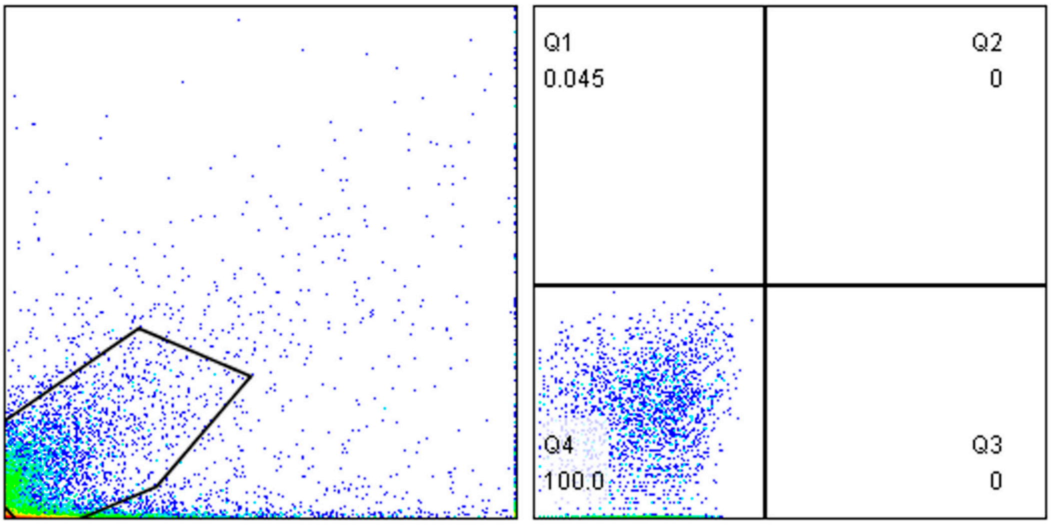

Supplementary Figure S3. **Available flow cytometry gating information for splenic T-cell subset analysis.** Splenocyte events were first selected using FSC/SSC gating to obtain lymphocyte-enriched events. The available unstained/negative control was used to establish the positive gates for fluorescence-positive populations, and the same gate settings were applied consistently to the analyzed samples.
